# The conclusion of a comparative efficacy study of fluralaner and sarolaner against the tick *Amblyomma americanum* on dogs is based on results obtained at study times that are outside the fluralaner label recommendations

**DOI:** 10.1186/s13071-017-2100-1

**Published:** 2017-03-24

**Authors:** Rob Armstrong

**Affiliations:** MSD Animal Health, 2 Giralda Farms, Madison, NJ 07940 USA

**Keywords:** Fluralaner, Sarolaner, *Ambloymma americanum*, Efficacy, Bravecto, Simparica

## Abstract

The only fluralaner-related conclusion presented in a study comparing the efficacy of fluralaner and sarolaner for control of the tick *Amblyomma americanum* on dogs is based on study times that are outside the label administration recommendations. Label recommendations for fluralaner treatment of *A. americanum* on dogs in the USA require re-administration at 56 days. This 56 day re-administration was not conducted in the study; therefore, all assessed time points following 56 days post-treatment in the study present comparisons that are not consistent with fluralaner administration recommendations. The only comparative time point assessed prior to 56 days showing a difference between treatments was at 42 days post-administration, a time point when methodological problems were identified by the investigators. Therefore, the only comparative study conclusion that a difference was shown between fluralaner and sarolaner beyond 6 weeks (42 days) after treatment is not based on recommended product use. Furthermore, if the study does not show that there is a difference between the treatments at times when the products are used as recommended, then there also can be no comparative discussion of the risk of tick-borne pathogen transmission risk between treatments.

## Letter to the Editor

A recent publication compares the efficacy of sarolaner and fluralaner in a challenge study on dogs with the tick *Amblyomma americanum* [1]. The study duration extends beyond 56 days; however, the investigators did not re-treat study dogs with fluralaner at day 56, as recommended for control of this tick on the product label in the USA [2] - the only country where this tick is included on the product label. Therefore, the investigators did not administer fluralaner (Bravecto, MSD Animal Health, Madison, NJ, USA) according to product label recommendations. This failure to follow label recommendations is noted in the discussion, but is not recorded in the abstract. In contrast, the comparative product sarolaner (Simparica, Zoetis, Florham Park, NJ, USA) was administered every 30 days throughout the study in accordance with its USA label recommendations [3].

The comparative results for the efficacy of the two treatments should only be presented during the period of the study when the products were used as recommended. Therefore, any comparative results beyond day 42 in this study (the last assessment point before the 8 week mark) are not based on the recommended use. Comparative results at 58 days post-administration were assessed at 2 days beyond the recommended fluralaner re-administration date, 48 hours after fluralaner should have been re-administered if the label recommendations were followed.

The results presented in the paper [1] show that all reported efficacies between the treatments are not significantly different until 42 days post-administration (except for a marginal significance of *P* = 0.044 at 12 h on day 0). At the 42 day time point both treatments had a low efficacy, and the authors report in the discussion that that the reason for this is unknown and may relate to a prolonged time between tick infestation and onset of tick feeding [1]. Therefore, it is quite possible that the reported difference at this time point is associated with a methodological problem.

The only conclusion presented in the paper that follows from the study objective of comparing the efficacy of fluralaner and sarolaner is that a difference is apparent from day 42 and later [1]. However, as shown above, the results at day 42 are associated with a methodological problem, and the comparisons beyond 42 days are assessed at a time when fluralaner use would not be as recommended on the product label. Therefore, the only comparative conclusion reached in the paper is not based on correct administration of fluralaner.

Furthermore, a number of one-sided statements are presented in the paper regarding tick-borne pathogen transmission, e.g. “the consistent efficacy of a single oral dose of sarolaner shown in this study should help to reduce the risk of a treated dog on a monthly treatment regime becoming infected with the pathogens transmitted by *A. americanum*” [1]. These one-sided statements are inappropriate because the results presented do not show a difference between the products when used as recommended.

## References

[1] Six RH, Young DR, Myers MR, Mahabir SP (2016). Comparative speed of kill of sarolaner (Simparica™ Chewables) and fluralaner (Bravecto®) against induced infestations of *Amblyomma americanum* on dogs. Parasit Vectors.

[2] FDA, FOI. NADA 141–426. BRAVECTO. http://www.fda.gov/downloads/animalveterinary/products/approvedanimaldrugproducts/foiadrugsummaries/ucm399075.pdf. Accessed 20 Aug 2016.

[3] FDA, FOI. NADA 141–452. SIMPARICA. http://www.fda.gov/downloads/AnimalVeterinary/Products/ApprovedAnimalDrugProducts/FOIADrugSummaries/UCM488816.pdf. Accessed 23 Aug 2016.

